# Supplementary material for: Characterization of Novel Trypanosoma cruzi-Specific Antigen with Potential Use in the Diagnosis of Chagas Disease
Source: Int J Mol Sci. 2024 Jan 18;25(2):1202. doi: 10.3390/ijms25021202 (PMC10816184; doi:10.3390/ijms25021202)

Figure S1. Reactivity of scFv 6B6 against *T. cruzi* and other trypanosomatids by inmmunoblot. A total of 30 µg of protein extracts from different parasites were resolved by SDS-PAGE, transferred to a nitrocellulose membrane, and assayed by WB using the antibodies scFv 6B6 (upper panel) and β-tubulin (loading control, lower panel). Tc: epimastigote *T. cruzi* Dm28c strain. Tb: *T. brucei.* B and P, bloodstream and procyclic form, respectively. Lm: promastigote *L. mexicana*. La: promastigote *L. amazonensis*. Li: promastigote *L. infantum*. Lb: promastigote *L. brazilensis*. Molecular weight markers (MM) (in kDa) are indicated on the left.

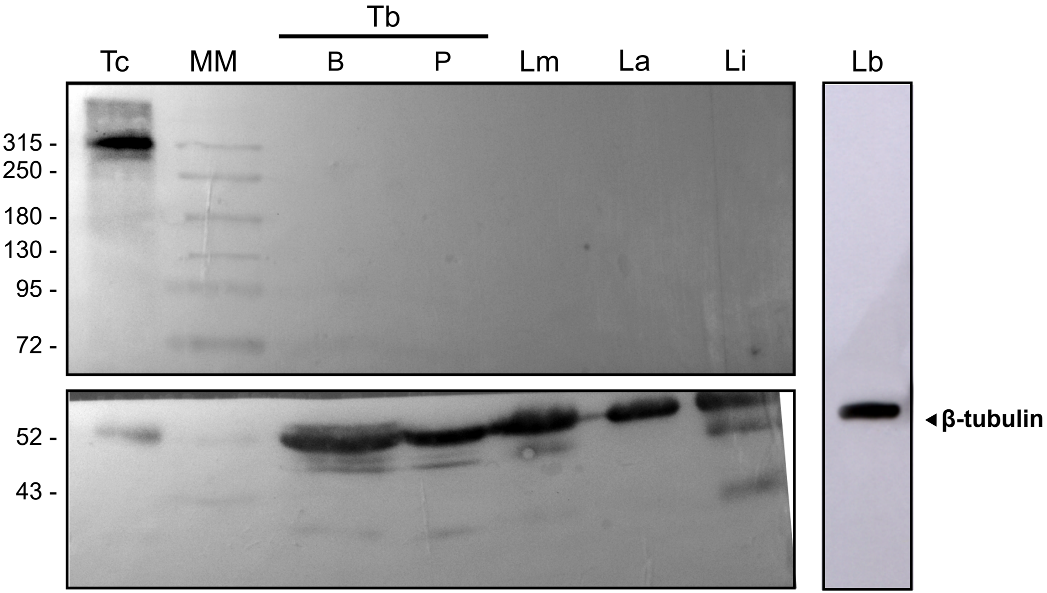

Supplement: Supplementary file 1 [file ijms-25-01202-s001.zip › Figure Supplementary 1 .pdf]
