# Supplementary material for: Characterization of Novel Trypanosoma cruzi-Specific Antigen with Potential Use in the Diagnosis of Chagas Disease
Source: Int J Mol Sci. 2024 Jan 18;25(2):1202. doi: 10.3390/ijms25021202 (PMC10816184; doi:10.3390/ijms25021202)

Figure S2. Reactivity of scFv 6B6 against mammalian tissues. Binding of scFv 6B6 against mouse organ tissue extracts or *T. cruzi* epimastigote lysate (TcE) was evaluated by ELISA. Data are expressed as OD450nm (means ± SD; n = 3). Differences between means of binding to different lysates vs. BSA were evaluated by one-way ANOVA followed by Dunn's multiple comparison tests. ****$p<0.0001$. Sm: skeletal muscle; Ln: lymph node; Ki: kidney; Bm: bone-marrow; Si: small intestine; Lv: liver; Sp: spleen; Ov: ovary; Ts: testis; He: heart; Wb: whole brain; Ob: olfactory bulb; Sc: spinal cord; Ce: cerebellum; Hs: hypothalamus; Hp: hippocampus; Md: midbrain; Cx: cortex; Sbm: striated muscle; Pg: pituitary gland; Lg: lung; Li: large intestine.

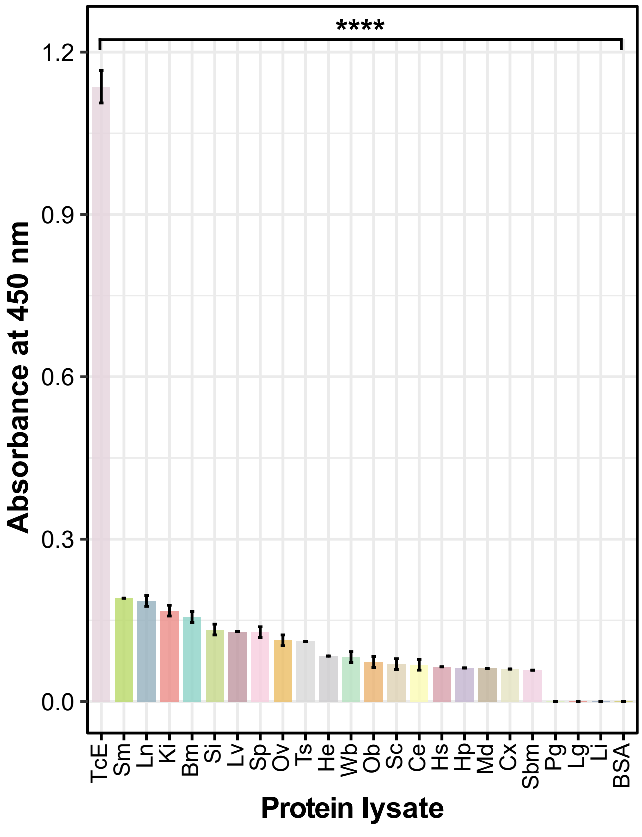

Supplement: Supplementary file 1 [file ijms-25-01202-s001.zip › Figure Supplementary 2.pdf]
