# Supplementary material for: Characterization of Novel Trypanosoma cruzi-Specific Antigen with Potential Use in the Diagnosis of Chagas Disease
Source: Int J Mol Sci. 2024 Jan 18;25(2):1202. doi: 10.3390/ijms25021202 (PMC10816184; doi:10.3390/ijms25021202)

Figure S3. Binding of full-length 6B6 antibody (chim m6B6) to Tc323. Western Blot analysis using chim m6B6 antibody. Total protein lysate from *T. cruzi* CL-Brener (Tc; 40 μg) and immunoprecipitated Tc323 protein (IP-Tc323; 10 μg) was subjected to SDS-PAGE (8%) gel, transferred to a nitrocellulose membrane, and incubated with chim m6B6. Molecular weight markers (in kDa) are indicated on the left.

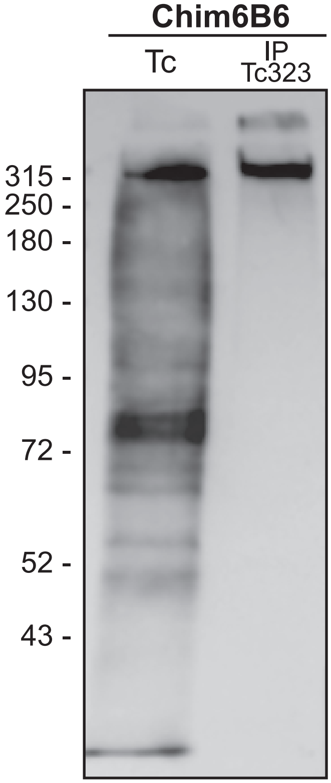

Supplement: Supplementary file 1 [file ijms-25-01202-s001.zip › Figure Supplementary 3.pdf]
