# Supplementary material for: Characterization of Novel Trypanosoma cruzi-Specific Antigen with Potential Use in the Diagnosis of Chagas Disease
Source: Int J Mol Sci. 2024 Jan 18;25(2):1202. doi: 10.3390/ijms25021202 (PMC10816184; doi:10.3390/ijms25021202)

Figure S4. Microsynteny analysis of the genomic regions harboring Tc323. A 38520 bp genomic fragment (*T. cruzi* Y strain) containing the Tc323 encoding gene (brown arrow) was compared with homologous regions from Esmeraldo like, Bug2148, Brazil, S231 T. cruzi strains and T. brucei TREU927.

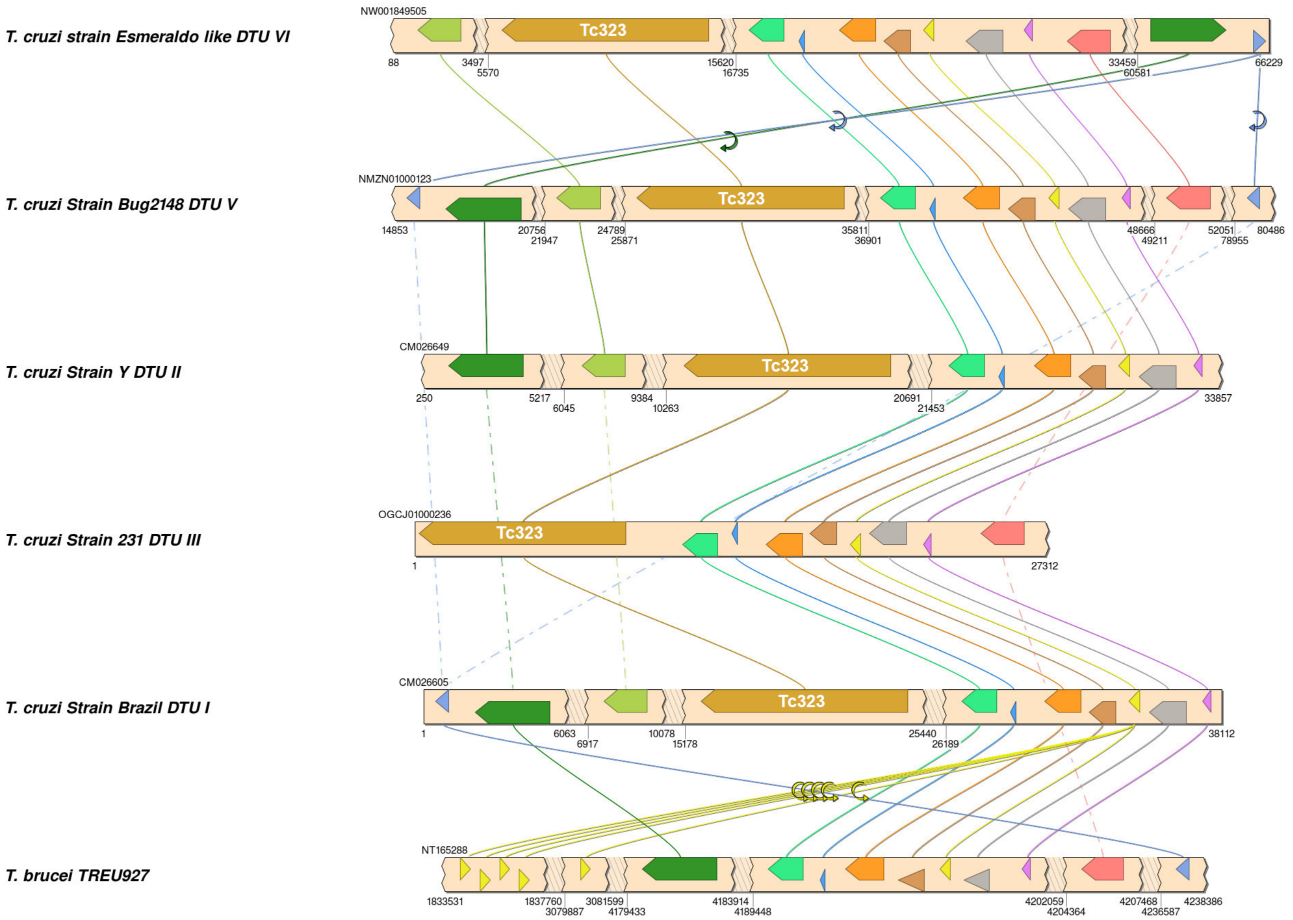

Supplement: Supplementary file 1 [file ijms-25-01202-s001.zip › Figure Supplementary 4.pdf]
