# Supplementary material for: Characterization of Novel Trypanosoma cruzi-Specific Antigen with Potential Use in the Diagnosis of Chagas Disease
Source: Int J Mol Sci. 2024 Jan 18;25(2):1202. doi: 10.3390/ijms25021202 (PMC10816184; doi:10.3390/ijms25021202)

Figure S5. Subcellular localization of Tc323 protein by immunofluoresence. Permeabilized epimastigote form of *T. cruzi* were labeled using chim m6B6, chim mA2R1, goat anti-mouse or goat anti-rabbit (red) and anti-BiP or anti-GRASP (green) antibodies. Nuclei and kinetoplasts were stained with DAPI (blue). Bars: 5 μm.

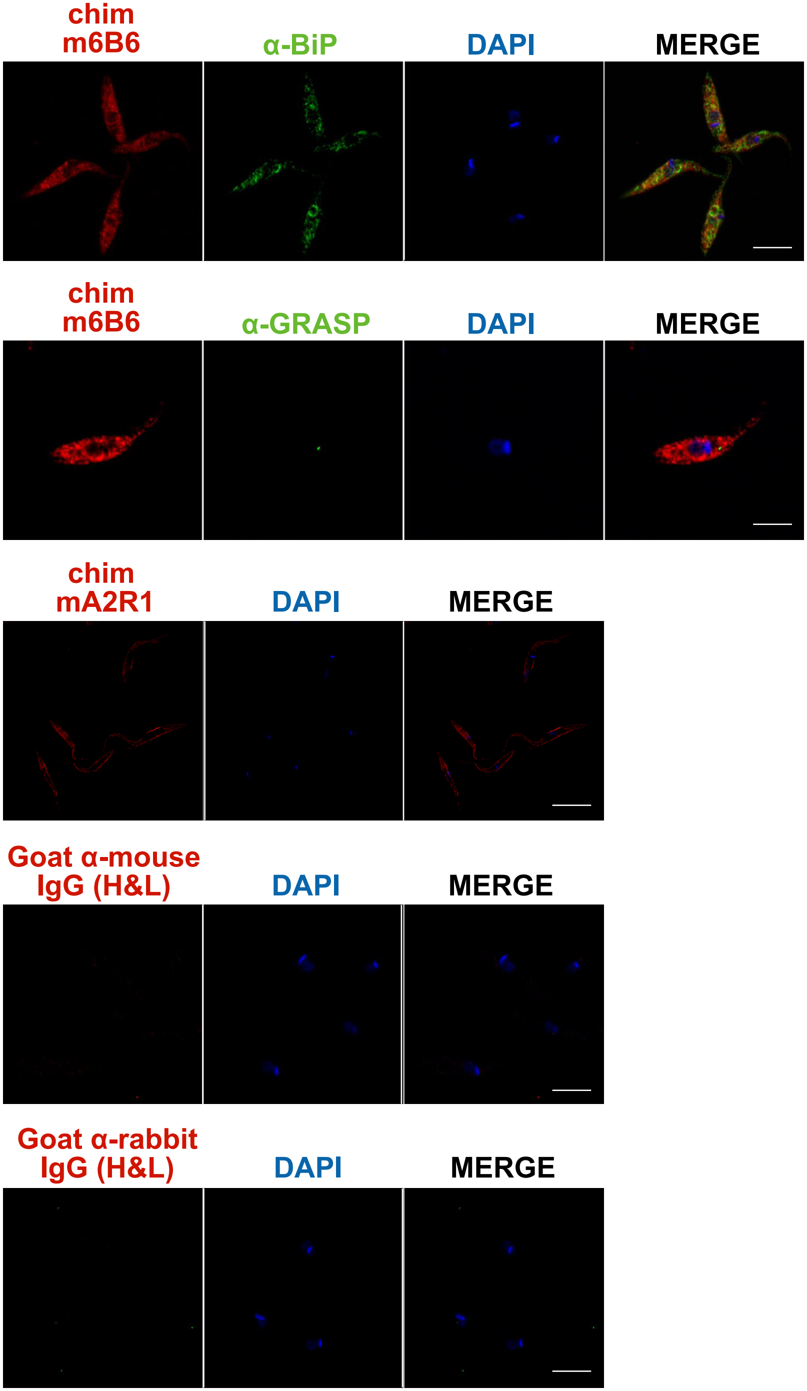

Supplement: Supplementary file 1 [file ijms-25-01202-s001.zip › Figure Supplementary 5.pdf]
