# Supplementary material for: Characterization of Novel Trypanosoma cruzi-Specific Antigen with Potential Use in the Diagnosis of Chagas Disease
Source: Int J Mol Sci. 2024 Jan 18;25(2):1202. doi: 10.3390/ijms25021202 (PMC10816184; doi:10.3390/ijms25021202)

Figure S6. Plasma reactivity against *T. cruzi* and *L. mexicana* protein lysate. Plasma from patients with chronic Chagas disease (CCD), cutaneous leishmaniasis (CL), *T. cruzi* and *Leishmania spp.* infection (mixed infection; MI) and non-infected individuals (NI) were tested for their reactivity against parasite lysate by ELISA. The OD_{450nm} is shown for the plasma samples at each of the various dilutions expressed as the inverse of the dilution factor. Each ELISA graph shows data from an individual subject.

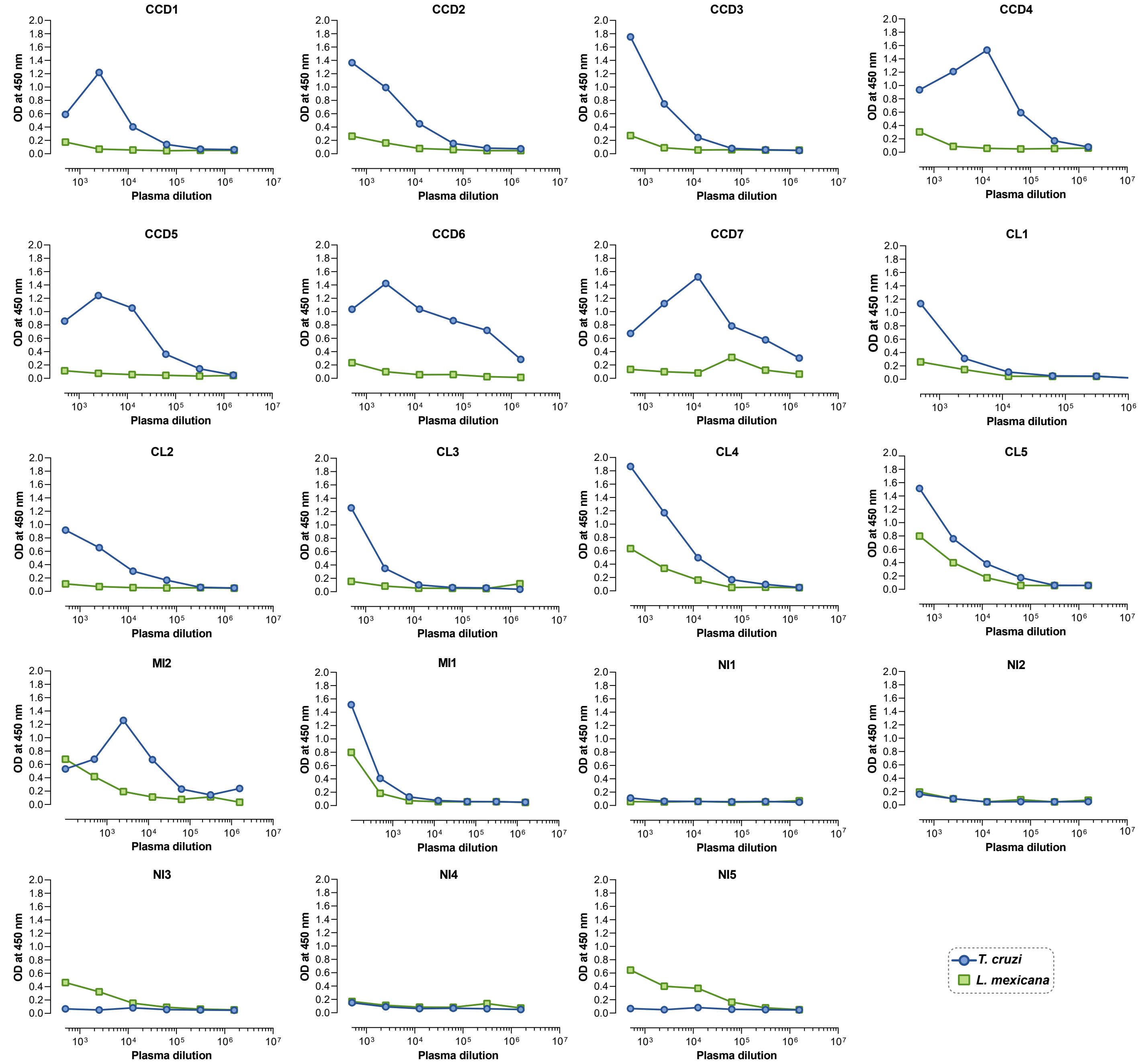

Supplement: Supplementary file 1 [file ijms-25-01202-s001.zip › Figure Supplementary 6.pdf]
